# Supplementary material for: A fault-intrusion-tolerant system and deadline-aware algorithm for scheduling scientific workflow in the cloud
Source: PeerJ Comput Sci. 2021 Nov 2;7:e747. doi: 10.7717/peerj-cs.747 (PMC8576554; doi:10.7717/peerj-cs.747)
Supplement: Supplemental Information 6 [file peerj-cs-07-747-s006.zip › Myproject1.1/docs/constant-values.html]

Constant Field Values


JavaScript is disabled on your browser.


- Overview
- Package
- Class
- Use
- Tree
- Deprecated
- Index
- Help

- Prev
- Next

- Frames
- No Frames

- All Classes

# Constant Field Values

## Contents

- org.cloudbus.\*
- org.workflowsim.\*

## org.cloudbus.\*

- org.cloudbus.cloudsim.Cloudlet

  | Modifier and Type | Constant Field | Value |
  | --- | --- | --- |
  |  |  |  |
  | --- | --- | --- |
  | `public static final int` | `CANCELED` | `6` |
  | `public static final int` | `CREATED` | `0` |
  | `public static final int` | `FAILED` | `5` |
  | `public static final int` | `FAILED_RESOURCE_UNAVAILABLE` | `9` |
  | `public static final int` | `INEXEC` | `3` |
  | `public static final int` | `PAUSED` | `7` |
  | `public static final int` | `QUEUED` | `2` |
  | `public static final int` | `READY` | `1` |
  | `public static final int` | `RESUMED` | `8` |
  | `public static final int` | `SUCCESS` | `4` |
- org.cloudbus.cloudsim.DatacenterCharacteristics

  | Modifier and Type | Constant Field | Value |
  | --- | --- | --- |
  |  |  |  |
  | --- | --- | --- |
  | `public static final int` | `ADVANCE_RESERVATION` | `4` |
  | `public static final int` | `OTHER_POLICY_DIFFERENT_RATING` | `3` |
  | `public static final int` | `OTHER_POLICY_SAME_RATING` | `2` |
  | `public static final int` | `SPACE_SHARED` | `1` |
  | `public static final int` | `TIME_SHARED` | `0` |
- org.cloudbus.cloudsim.DataCloudTags

  | Modifier and Type | Constant Field | Value |
  | --- | --- | --- |
  |  |  |  |
  | --- | --- | --- |
  | `public static final int` | `CTLG_ADD_MASTER` | `610` |
  | `public static final int` | `CTLG_ADD_MASTER_ERROR` | `613` |
  | `public static final int` | `CTLG_ADD_MASTER_ERROR_FULL` | `614` |
  | `public static final int` | `CTLG_ADD_MASTER_RESULT` | `611` |
  | `public static final int` | `CTLG_ADD_MASTER_SUCCESSFUL` | `612` |
  | `public static final int` | `CTLG_ADD_REPLICA` | `630` |
  | `public static final int` | `CTLG_ADD_REPLICA_ERROR` | `633` |
  | `public static final int` | `CTLG_ADD_REPLICA_ERROR_DOESNT_EXIST` | `634` |
  | `public static final int` | `CTLG_ADD_REPLICA_ERROR_FULL` | `635` |
  | `public static final int` | `CTLG_ADD_REPLICA_RESULT` | `631` |
  | `public static final int` | `CTLG_ADD_REPLICA_SUCCESSFUL` | `632` |
  | `public static final int` | `CTLG_DELETE_MASTER` | `620` |
  | `public static final int` | `CTLG_DELETE_MASTER_DOESNT_EXIST` | `624` |
  | `public static final int` | `CTLG_DELETE_MASTER_ERROR` | `623` |
  | `public static final int` | `CTLG_DELETE_MASTER_REPLICAS_EXIST` | `625` |
  | `public static final int` | `CTLG_DELETE_MASTER_RESULT` | `621` |
  | `public static final int` | `CTLG_DELETE_MASTER_SUCCESSFUL` | `622` |
  | `public static final int` | `CTLG_DELETE_REPLICA` | `640` |
  | `public static final int` | `CTLG_DELETE_REPLICA_ERROR` | `643` |
  | `public static final int` | `CTLG_DELETE_REPLICA_ERROR_DOESNT_EXIST` | `644` |
  | `public static final int` | `CTLG_DELETE_REPLICA_RESULT` | `641` |
  | `public static final int` | `CTLG_DELETE_REPLICA_SUCCESSFUL` | `642` |
  | `public static final int` | `CTLG_FILE_ATTR_DELIVERY` | `606` |
  | `public static final int` | `CTLG_FILTER` | `607` |
  | `public static final int` | `CTLG_FILTER_DELIVERY` | `608` |
  | `public static final int` | `CTLG_GET_FILE_ATTR` | `605` |
  | `public static final int` | `CTLG_GET_REPLICA` | `601` |
  | `public static final int` | `CTLG_GET_REPLICA_LIST` | `603` |
  | `public static final int` | `CTLG_MODIFY_MASTER` | `650` |
  | `public static final int` | `CTLG_MODIFY_MASTER_ERROR` | `653` |
  | `public static final int` | `CTLG_MODIFY_MASTER_ERROR_DOESNT_EXIST` | `654` |
  | `public static final int` | `CTLG_MODIFY_MASTER_ERROR_READ_ONLY` | `655` |
  | `public static final int` | `CTLG_MODIFY_MASTER_RESULT` | `651` |
  | `public static final int` | `CTLG_MODIFY_MASTER_SUCCESSFUL` | `652` |
  | `public static final int` | `CTLG_REPLICA_DELIVERY` | `602` |
  | `public static final int` | `CTLG_REPLICA_LIST_DELIVERY` | `604` |
  | `public static final int` | `DATAcloudlet_SUBMIT` | `406` |
  | `public static final int` | `DEFAULT_MTU` | `1500` |
  | `public static final int` | `DEFAULT_STORAGE_SIZE` | `10000000` |
  | `public static final int` | `FILE_ADD_ERROR` | `524` |
  | `public static final int` | `FILE_ADD_ERROR_ACCESS_DENIED` | `525` |
  | `public static final int` | `FILE_ADD_ERROR_EMPTY` | `522` |
  | `public static final int` | `FILE_ADD_ERROR_EXIST_READ_ONLY` | `523` |
  | `public static final int` | `FILE_ADD_ERROR_STORAGE_FULL` | `521` |
  | `public static final int` | `FILE_ADD_MASTER` | `510` |
  | `public static final int` | `FILE_ADD_MASTER_RESULT` | `511` |
  | `public static final int` | `FILE_ADD_REPLICA` | `512` |
  | `public static final int` | `FILE_ADD_REPLICA_RESULT` | `513` |
  | `public static final int` | `FILE_ADD_SUCCESSFUL` | `520` |
  | `public static final int` | `FILE_DELETE_ERROR` | `541` |
  | `public static final int` | `FILE_DELETE_ERROR_ACCESS_DENIED` | `545` |
  | `public static final int` | `FILE_DELETE_ERROR_DOESNT_EXIST` | `543` |
  | `public static final int` | `FILE_DELETE_ERROR_IN_USE` | `544` |
  | `public static final int` | `FILE_DELETE_ERROR_READ_ONLY` | `542` |
  | `public static final int` | `FILE_DELETE_MASTER` | `530` |
  | `public static final int` | `FILE_DELETE_MASTER_RESULT` | `531` |
  | `public static final int` | `FILE_DELETE_REPLICA` | `532` |
  | `public static final int` | `FILE_DELETE_REPLICA_RESULT` | `533` |
  | `public static final int` | `FILE_DELETE_SUCCESSFUL` | `540` |
  | `public static final int` | `FILE_DELIVERY` | `502` |
  | `public static final int` | `FILE_MODIFY` | `550` |
  | `public static final int` | `FILE_MODIFY_ERROR` | `561` |
  | `public static final int` | `FILE_MODIFY_ERROR_ACCESS_DENIED` | `565` |
  | `public static final int` | `FILE_MODIFY_ERROR_DOESNT_EXIST` | `563` |
  | `public static final int` | `FILE_MODIFY_ERROR_IN_USE` | `564` |
  | `public static final int` | `FILE_MODIFY_ERROR_READ_ONLY` | `562` |
  | `public static final int` | `FILE_MODIFY_RESULT` | `551` |
  | `public static final int` | `FILE_MODIFY_SUCCESSFUL` | `560` |
  | `public static final int` | `FILE_REQUEST` | `501` |
  | `public static final int` | `INQUIRY_GLOBAL_RC_LIST` | `403` |
  | `public static final int` | `INQUIRY_LOCAL_RC_LIST` | `402` |
  | `public static final int` | `INQUIRY_RC_LIST` | `404` |
  | `public static final int` | `INQUIRY_RC_RESULT` | `405` |
  | `public static final int` | `PKT_SIZE` | `150000` |
  | `public static final int` | `REGISTER_REPLICA_CTLG` | `401` |
- org.cloudbus.cloudsim.File

  | Modifier and Type | Constant Field | Value |
  | --- | --- | --- |
  |  |  |  |
  | --- | --- | --- |
  | `public static final int` | `NOT_REGISTERED` | `-1` |
  | `public static final int` | `TYPE_RAW_DATA` | `1` |
  | `public static final int` | `TYPE_RECONSTRUCTED_DATA` | `2` |
  | `public static final int` | `TYPE_TAG_DATA` | `3` |
  | `public static final int` | `TYPE_UNKOWN` | `0` |
- org.cloudbus.cloudsim.Pe

  | Modifier and Type | Constant Field | Value |
  | --- | --- | --- |
  |  |  |  |
  | --- | --- | --- |
  | `public static final int` | `BUSY` | `2` |
  | `public static final int` | `FAILED` | `3` |
  | `public static final int` | `FREE` | `1` |

- org.cloudbus.cloudsim.core.CloudSimTags

  | Modifier and Type | Constant Field | Value |
  | --- | --- | --- |
  |  |  |  |
  | --- | --- | --- |
  | `public static final int` | `ABRUPT_END_OF_SIMULATION` | `-2` |
  | `public static final int` | `CLOUDLET_CANCEL` | `23` |
  | `public static final int` | `CLOUDLET_MOVE` | `29` |
  | `public static final int` | `CLOUDLET_MOVE_ACK` | `30` |
  | `public static final int` | `CLOUDLET_PAUSE` | `25` |
  | `public static final int` | `CLOUDLET_PAUSE_ACK` | `26` |
  | `public static final int` | `CLOUDLET_RESUME` | `27` |
  | `public static final int` | `CLOUDLET_RESUME_ACK` | `28` |
  | `public static final int` | `CLOUDLET_RETURN` | `20` |
  | `public static final int` | `CLOUDLET_STATUS` | `24` |
  | `public static final int` | `CLOUDLET_SUBMIT` | `21` |
  | `public static final int` | `CLOUDLET_SUBMIT_ACK` | `22` |
  | `public static final int` | `DEFAULT_BAUD_RATE` | `9600` |
  | `public static final int` | `END_OF_SIMULATION` | `-1` |
  | `public static final int` | `EXPERIMENT` | `1` |
  | `public static final int` | `FALSE` | `0` |
  | `public static final int` | `INFOPKT_RETURN` | `106` |
  | `public static final int` | `INFOPKT_SUBMIT` | `105` |
  | `public static final int` | `INSIGNIFICANT` | `0` |
  | `public static final int` | `Network_Event_DOWN` | `46` |
  | `public static final int` | `Network_Event_Host` | `47` |
  | `public static final int` | `Network_Event_send` | `44` |
  | `public static final int` | `Network_Event_UP` | `43` |
  | `public static final int` | `NextCycle` | `48` |
  | `public static final int` | `RECORD_STATISTICS` | `10` |
  | `public static final int` | `REGISTER_REGIONAL_GIS` | `13` |
  | `public static final int` | `REGISTER_RESOURCE` | `2` |
  | `public static final int` | `REGISTER_RESOURCE_AR` | `3` |
  | `public static final int` | `REQUEST_REGIONAL_GIS` | `14` |
  | `public static final int` | `RESOURCE_AR_LIST` | `5` |
  | `public static final int` | `RESOURCE_CHARACTERISTICS` | `6` |
  | `public static final int` | `RESOURCE_CHARACTERISTICS_REQUEST` | `15` |
  | `public static final int` | `RESOURCE_DYNAMICS` | `7` |
  | `public static final int` | `RESOURCE_LIST` | `4` |
  | `public static final int` | `RESOURCE_NUM_FREE_PE` | `9` |
  | `public static final int` | `RESOURCE_NUM_PE` | `8` |
  | `public static final int` | `RESOURCE_Register` | `45` |
  | `public static final int` | `RETURN_ACC_STATISTICS_BY_CATEGORY` | `12` |
  | `public static final int` | `RETURN_STAT_LIST` | `11` |
  | `public static final double` | `SCHEDULE_NOW` | `0.0` |
  | `public static final int` | `TRUE` | `1` |
  | `public static final int` | `VM_BROKER_EVENT` | `42` |
  | `public static final int` | `VM_CREATE` | `31` |
  | `public static final int` | `VM_CREATE_ACK` | `32` |
  | `public static final int` | `VM_DATA_ADD` | `37` |
  | `public static final int` | `VM_DATA_ADD_ACK` | `38` |
  | `public static final int` | `VM_DATA_DEL` | `39` |
  | `public static final int` | `VM_DATA_DEL_ACK` | `40` |
  | `public static final int` | `VM_DATACENTER_EVENT` | `41` |
  | `public static final int` | `VM_DESTROY` | `33` |
  | `public static final int` | `VM_DESTROY_ACK` | `34` |
  | `public static final int` | `VM_MIGRATE` | `35` |
  | `public static final int` | `VM_MIGRATE_ACK` | `36` |
- org.cloudbus.cloudsim.core.SimEntity

  | Modifier and Type | Constant Field | Value |
  | --- | --- | --- |
  |  |  |  |
  | --- | --- | --- |
  | `public static final int` | `FINISHED` | `3` |
  | `public static final int` | `HOLDING` | `2` |
  | `public static final int` | `RUNNABLE` | `0` |
  | `public static final int` | `WAITING` | `1` |
- org.cloudbus.cloudsim.core.SimEvent

  | Modifier and Type | Constant Field | Value |
  | --- | --- | --- |
  |  |  |  |
  | --- | --- | --- |
  | `public static final int` | `CREATE` | `3` |
  | `public static final int` | `ENULL` | `0` |
  | `public static final int` | `HOLD_DONE` | `2` |
  | `public static final int` | `SEND` | `1` |

- org.cloudbus.cloudsim.examples.power.Constants

  | Modifier and Type | Constant Field | Value |
  | --- | --- | --- |
  |  |  |  |
  | --- | --- | --- |
  | `public static final int` | `CLOUDLET_LENGTH` | `216000000` |
  | `public static final int` | `CLOUDLET_PES` | `1` |
  | `public static final boolean` | `ENABLE_OUTPUT` | `true` |
  | `public static final int` | `HOST_BW` | `1000000` |
  | `public static final int` | `HOST_STORAGE` | `1000000` |
  | `public static final int` | `HOST_TYPES` | `2` |
  | `public static final boolean` | `OUTPUT_CSV` | `false` |
  | `public static final double` | `SCHEDULING_INTERVAL` | `300.0` |
  | `public static final double` | `SIMULATION_LIMIT` | `86400.0` |
  | `public static final int` | `VM_BW` | `100000` |
  | `public static final int` | `VM_SIZE` | `2500` |
  | `public static final int` | `VM_TYPES` | `4` |

- org.cloudbus.cloudsim.examples.power.planetlab.PlanetLabConstants

  | Modifier and Type | Constant Field | Value |
  | --- | --- | --- |
  |  |  |  |
  | --- | --- | --- |
  | `public static final int` | `NUMBER_OF_HOSTS` | `800` |

- org.cloudbus.cloudsim.examples.power.random.RandomConstants

  | Modifier and Type | Constant Field | Value |
  | --- | --- | --- |
  |  |  |  |
  | --- | --- | --- |
  | `public static final long` | `CLOUDLET_UTILIZATION_SEED` | `1L` |
  | `public static final int` | `NUMBER_OF_HOSTS` | `50` |
  | `public static final int` | `NUMBER_OF_VMS` | `50` |

- org.cloudbus.cloudsim.network.datacenter.AppCloudlet

  | Modifier and Type | Constant Field | Value |
  | --- | --- | --- |
  |  |  |  |
  | --- | --- | --- |
  | `public static final int` | `APP_MC` | `1` |
  | `public static final int` | `APP_Workflow` | `3` |
- org.cloudbus.cloudsim.network.datacenter.NetworkConstants

  | Modifier and Type | Constant Field | Value |
  | --- | --- | --- |
  |  |  |  |
  | --- | --- | --- |
  | `public static final int` | `Agg_LEVEL` | `1` |
  | `public static final int` | `COMMUNICATION_LENGTH` | `1` |
  | `public static final int` | `EDGE_LEVEL` | `2` |
  | `public static final int` | `EXECUTION` | `0` |
  | `public static final int` | `FILE_SIZE` | `300` |
  | `public static final int` | `FINISH` | `-2` |
  | `public static final int` | `OUTPUT_SIZE` | `300` |
  | `public static final int` | `PES_NUMBER` | `4` |
  | `public static final int` | `ROOT_LEVEL` | `0` |
  | `public static final int` | `WAIT_RECV` | `2` |
  | `public static final int` | `WAIT_SEND` | `1` |

- org.cloudbus.cloudsim.power.PowerVm

  | Modifier and Type | Constant Field | Value |
  | --- | --- | --- |
  |  |  |  |
  | --- | --- | --- |
  | `public static final int` | `HISTORY_LENGTH` | `30` |


## org.workflowsim.\*

- org.workflowsim.WorkflowSimTags

  | Modifier and Type | Constant Field | Value |
  | --- | --- | --- |
  |  |  |  |
  | --- | --- | --- |
  | `public static final int` | `CLOUDLET_CHECK` | `1006` |
  | `public static final int` | `CLOUDLET_UPDATE` | `1005` |
  | `public static final int` | `JOB_SUBMIT` | `1001` |
  | `public static final int` | `START_SIMULATION` | `1000` |
  | `public static final int` | `VM_STATUS_BUSY` | `1003` |
  | `public static final int` | `VM_STATUS_IDLE` | `1004` |
  | `public static final int` | `VM_STATUS_READY` | `1002` |

- org.workflowsim.utils.Parameters

  | Modifier and Type | Constant Field | Value |
  | --- | --- | --- |
  |  |  |  |
  | --- | --- | --- |
  | `public static final int` | `BASE` | `0` |

- Overview
- Package
- Class
- Use
- Tree
- Deprecated
- Index
- Help

- Prev
- Next

- Frames
- No Frames

- All Classes
